# Identification of a Soybean *MOTHER OF FT AND TFL1* Homolog Involved in Regulation of Seed Germination

**DOI:** 10.1371/journal.pone.0099642

**Published:** 2014-06-16

**Authors:** Qing Li, Chengming Fan, Xiaomei Zhang, Xu Wang, Faqiang Wu, Ruibo Hu, Yongfu Fu

**Affiliations:** 1 MOA Key Lab of Soybean Biology (Beijing), National Key Facility of Crop Gene Resource and Genetic Improvement, Institute of Crop Sciences, Chinese Academy of Agricultural Sciences, Beijing, China; 2 Institute of Genetics and Developmental Biology, Chinese Academy of Sciences, Beijing, China; 3 CAS Key Laboratory of Biofuels, Shandong Provincial Key Laboratory of Energy Genetics, Qingdao Institute of Bioenergy and Bioprocess Technology, Chinese Academy of Sciences, Qingdao, China; China Agricultural University, China

## Abstract

Seed germination is an important event in the life cycle of seed plants, and is controlled by complex and coordinated genetic networks. Many genes involved in the regulation of this process have been identified in different plant species so far. Recent studies in both *Arabidopsis* and wheat have uncovered a new role of *MOTHER OF FT AND TFL1* (*MFT*) in seed germination. Here, we reported a homolog of *MFT* in soybean (*GmMFT*) which strongly expressed in seeds. Detailed expression analysis showed that the mRNA level of *GmMFT* increased with seed development but declined during seed germination. The transcription of *GmMFT* also responded to exogenous application of ABA and GA3. Ectopic expression of *GmMFT* CDS in *Arabidopsis* moderately inhibited seed germination. All these evidences suggest that *GmMFT* may be a negative regulator of seed germination.

## Introduction

Seed germination, a key ecological and agronomic trait for seed plants, determines when plants enter natural or agricultural ecosystems and marks the beginning of a new growth cycle [1]. It is controlled by both intrinsic and environmental cues, which are mainly regulated by two antagonistic phytohormones, abscisic acid (ABA) and gibberellin (GA) [2]. ABA is a negative regulator of seed germination, while GA promotes the completion of germination, counteracting the effects of ABA [3].

To date, many loci that are involved in the ABA or GA-associated regulation of seed germination have been identified. The significance of ABA biosynthesis in seed germination can be seen in the altered germination frequencies of ABA biosynthesis mutants, such as *aba1*, *aba2*, *aba3, nced6*, *nced9* and *aao3* [3]–[7]. The seeds of *aba1*, *aba2*, *aba3* and *aao3* showed reduced dormancy and their germination were resistant to the gibberellin biosynthesis inhibitor [4]–[7]. The *nced6/nced9* double mutant also exhibited decreased dormancy and increased germination, but not in single mutant seeds [3]. The hormonal action of ABA in plants is controlled by the precise balance between its biosynthesis and catabolism. Thus, catabolism of ABA is important in regulating germination potential. *CYP707A* gene family (*CYP707A1*–*4*), which encode ABA 8′-hydroxylation, are shown to play the predominant role in ABA catabolism. Among them, *CYP707A1* and *CYP707A2* are indispensable for proper control of seed dormancy and germination [8], [9]. In addition to ABA content, ABA enhances seed dormancy and inhibits seed germination through various signaling components, including *ABA-INSENSITIVE3* (*ABI3*) and *ABI5* [10]–[14].

The germination-inhibiting effect of ABA is counteracted by GA. The significance of GA metabolic genes in germination can be seen in the altered germination rates of GA metabolic mutants, such as *ga20ox1* (formerly *ga5*), *ga3ox1* (formerly *ga4*), *ga3ox2* and *ga2ox2* [15]–[19]. Study of the function of GA biosynthesis in regulation of germination has identified a key enzyme named gibberellin 3-oxidase (GA3OX). This enzyme is encoded by four genes (*GA3OX1–4*), among which *GA3OX1* and *GA3OX2* are important for GA-regulated germination [17], [18]. The *ga3ox1*/*ga3ox2* double mutant displayed a severe defect in seed germination [18]. In contrast, mutants defective in GA 2-oxidases (GA2ox), which deactivate bioactive GA, showed reduced seed dormancy and partly promoted the seed germination during dark imbibition [19]. DELLA proteins are negative GA signaling components that inhibit different GA responses, including seed germination, stem elongation, and floral development [20]–[25]. There are five DELLA proteins in *Arabidopsis* (GAI, RGA, RGL1, RGL2, and RGL3), among which RGA, GAI, especially RGL2 play important roles in inhibiting seed germination [26]–[28].

*MOTHER OF FT AND TFL1* (*MFT*) belongs to the phosphatidyl ethanolamine-binding protein (PEBP) family, and is evolutionarily conserved in a wide range of multicellular land plants [29]. Based on sequence phylogenetic analysis, PEBP family genes in angiosperms can be divided into three subfamilies: *FT*-like, *TFL1*-like and *MFT*-like clades [30], and *MFT*-like clade is generally thought as the evolutionary ancestor to the other two clades [29]. Both *FT*-like and *TFL*-like genes are key regulators in floral initiation, but with reverse affects: *FT* promotes flowering, while *TFL* represses it [31]–[37]. The function of *MFT*-like genes is less characterized than the other two clades. Overexpression of *Arabidopsis MFT* (*AtMFT*) resulted in a slightly early flowering phenotype [38], while its loss-of-function mutants had no obvious changes on flowering time [14], [38]. Besides the role in flowering regulation, *MFT*-like genes were also reported to regulate seed dormancy and germination [14], [39], [40], which was in agreement with their seed-specific expression pattern [14], [30], [41], [42]. *AtMFT* has been shown to increase dormancy during seed development [40] and to promote germination in after-ripened imbibed seeds with exogenous ABA [14]. However, *Triticum aestivum MFT* (*TaMFT*) acted as a positive regulator of seed dormancy and inhibited seed germination [39].

In this study, we identified a soybean homolog of *MFT* (*GmMFT*). Our study showed that *GmMFT* mainly expressed in soybean seeds. Its expression increased during seed development, whereas decreased during seed germination. The expression of *GmMFT* also responded to both ABA and GA3 during seed germination. Ectopic expression of *GmMFT* in *Arabidopsis* inhibited the seed germination at the early stage. Taken together, we proposed that GmMFT was a potential negative regulator in seed germination.

## Results

### An *MFT* homolog is identified in soybean

Glyma05g34030 was identified as a soybean *MFT*-like gene by searching in Phytozome database (http://www.phytozome.org) with *Arabidopsis* MFT protein as bait, and was named as *GmMFT*. Its coding sequence (CDS) was then cloned from the cDNA of soybean cultivar Kennong 18. The resulting sequence was the same as the predicted Glyma05g34030 CDS.

**Figure 1:**
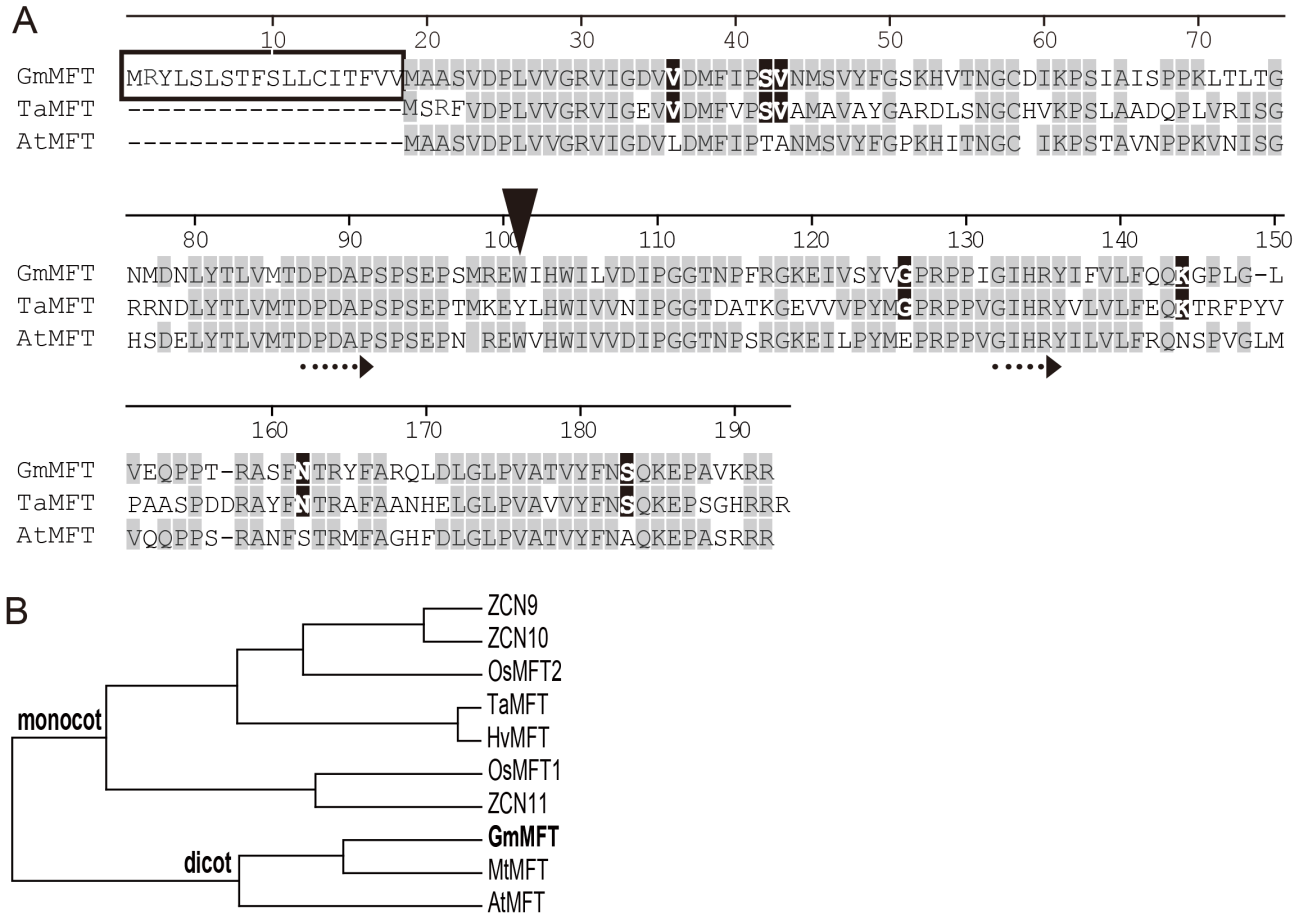
Bioinformatics analysis of MFT-like proteins. A, Alignment of amino acid sequences of MFT-like proteins in soybean, wheat, *Arabidopsis*. The black box indicates N-terminus extension of GmMFT; White letters in black background indicate the identical amino acid residues between only soybean and wheat; Black arrow indicates His/Tyr residue involved in FT/TFL1 function switching; Dotted arrows indicate conserved D-P-D-x-P and G-x-H-R motifs of MFT proteins. B, Phylogenetic analysis of MFT-like proteins. A neighbor-joining tree was constructed with MEGA 4.0 software based on the amino acid sequences. Bootstrap values were provided to show reliability at each node. The sequences are from *Zea mays* (ZCN9, ABX11011; ZCN10, ABW96233; ZCN11, ABX11013), *Oryza sativa* (OsMFT1, Os06g30370; OsMFT2, Os01g02120), *Triticum aestivum* (TaMFT, BAK78908), *Hordeum vulgare* (HvMFT, BAH24198), *Arabidopsis* (AtMFT, At1g18100), *Glycine max* (GmMFT, Glyma05g34030), *Medicago truncatula* (MtMFT, Medtr8g106840).

Because two *MFT* homologs from *Arabidopsis* and wheat were best studied functionally, we carried out amino acid sequence alignment of these three MFT-like proteins (GmMFT, TaMFT, and AtMFT) (Figure 1A). The result showed that sequences of these proteins were conserved and contained MFT typical motif or amino acid residues [29]. Furthermore, several amino acid residues were identical only between GmMFT and TaMFT or GmMFT and AtMFT. The key residue, which involved in distinguishing FT/TFL1 function on flowering regulation [31], was W (Trp) in GmMFT and AtMFT, instead of Y (Tyr in FT) or H (His in TFL1), leading to the hypothesis that GmMFT may have weak or no regulatory activity of flowering. It is noteworthy that there was a short extension of 18 amino acid residues at the N-terminal of GmMFT (Figure 1A). SignalP prediction suggested that this extension may serve as a signal peptide (Figure S1) [43]. Not surprisingly, GmMFT showed stronger homology to AtMFT than TaMFT (Figure S2), and was grouped into the dicot MFT-like clade in phylogenetic tree (Figure 1B).

### The expression pattern of *GmMFT*

**Figure 2:**
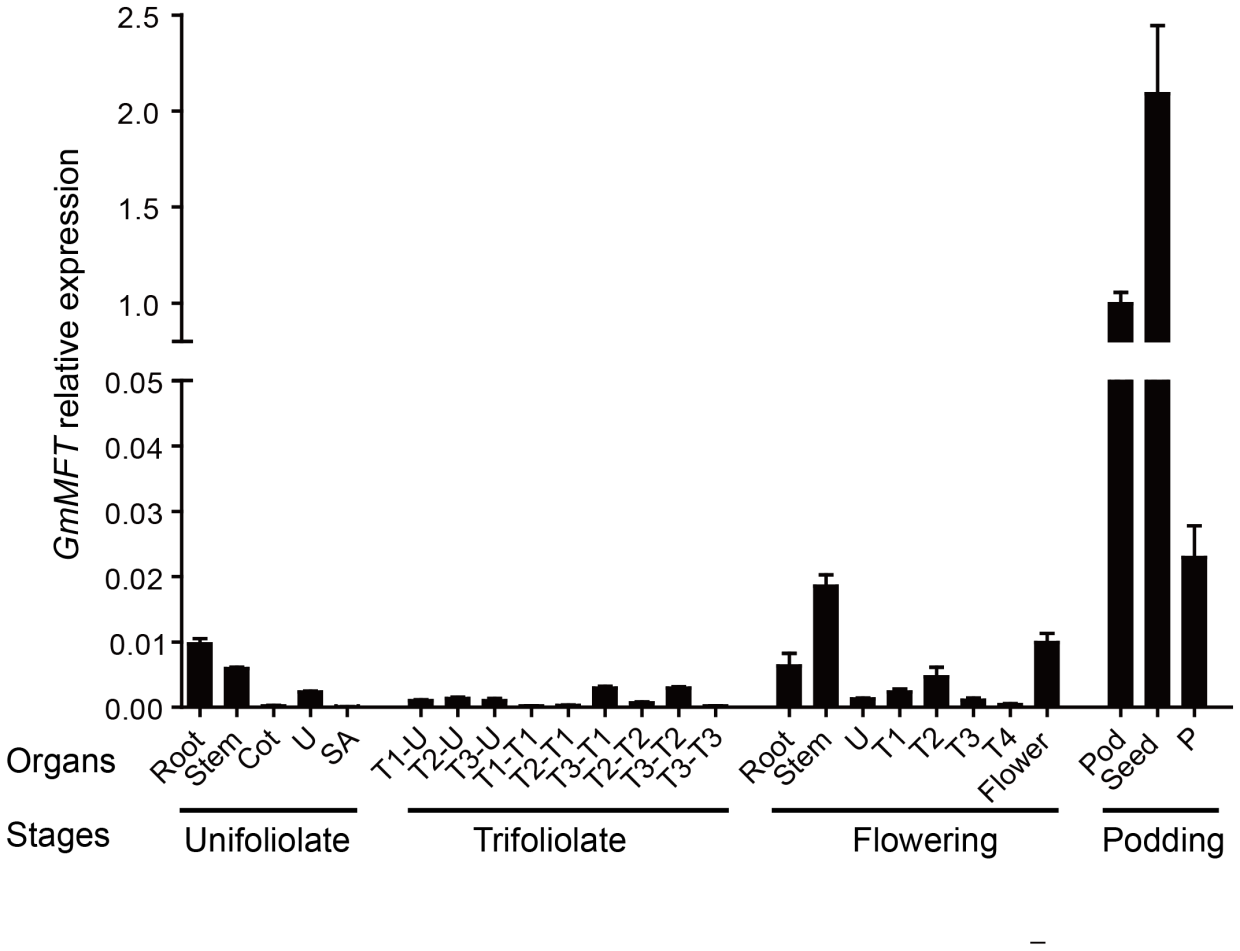
The expression pattern of *GmMFT* in different tissues/organs. Stages were defined as follows: Unifoliolate, unifoliolates fully opened; Trifoliolate, from 1^st^ trifoliolates fully opened to onset of flowering; Flowering, onset of flowering; Podding, initiation of pod growth. Abbreviations for tissues/organs: unifoliolate (U), cotyledon (Cot), shoot apex (SA), trifoliolate (T), pod without seeds (P). Tx means x^th^ trifoliolate. Tx-U represents unifoliolate at the x^th^ trifoliolate stage. For example, T3-U means unifoliolate at the 3^rd^ trifoliolate stage. Similarly, Tx-Tx represents x^th^ trifoliolate at the x^th^ trifoliolate stage. Three genes (*ACT11*, *UKN1*and *UKN2*) in combination were used for internal control. Error bars denoted SD.

To gain insight into the potential functions of *GmMFT*, its transcription profiles were analyzed by RT-qPCR (Figure 2). As shown in figure 2, the *GmMFT* transcripts were detected almost in all examined tissues/organs. Its expression was extremely high in seeds, relatively strong in pods, but very low in other tissues including dissected pods without seeds. This expression pattern of *GmMFT* was consistent with that of *AtMFT* [14], implying a possible role of *GmMFT* in seed dormancy or germination.

**Figure 3:**
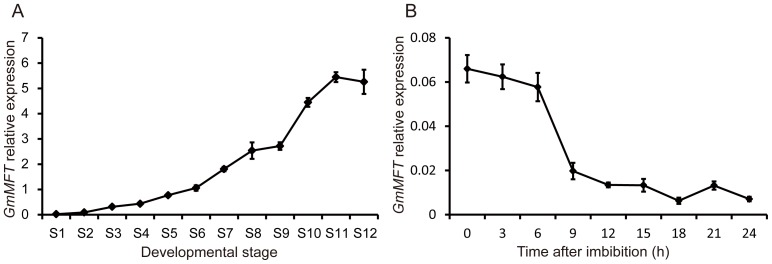
The expression pattern of *GmMFT* in developmental and germinating seeds. A, The *GmMFT* transcripts in seeds during seed development. Seed development was divided into 12 stages (S1–S12) based on the seed length as previously reported. B, The *GmMFT* transcripts in seeds during seed germination. Three genes (*ACT11*, *UKN1*and *UKN2*) in combination were used for internal control. Error bars denoted SD.

To test our speculation, the temporal expression pattern of *GmMFT* in seeds during seed development and germination was investigated (Figure 3). The progress of seed development was divided into 12 stages (S1–S12) based on seed length as previously reported [44]. Our result showed that *GmMFT* expression increased slowly during early seed developmental stage (S1–S5), and then increased rapidly during middle (S6–S10) and late (S11) stage (Figure 3A). When seed imbibition, the *GmMFT* transcripts continued to decline until seeds began to germinate (12 h after imbibition), and then kept at a relatively stable level (Figure 3B). Taken together, this expression pattern of *GmMFT*, which was increased during seed development and decreased during seed germination, further suggests that *GmMFT* may be involved in the control of seed dormancy or germination.

**Figure 4:**
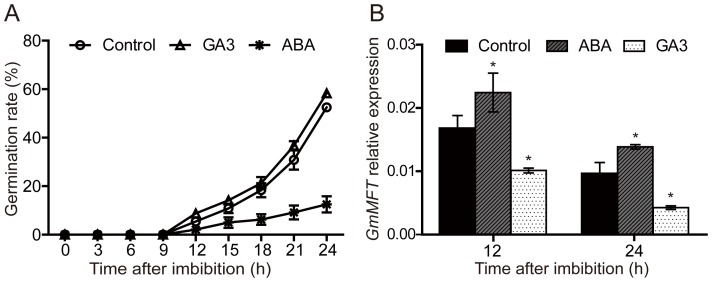
The expression pattern of *GmMFT* in germinating seeds with the treatments by exogenous ABA and GA3. A, The germination rates of soybean seeds under 10 µM ABA or 5 µM GA3 treatment; B, Relative expression of *GmMFT* in soybean seeds at 12 h, 24 h after treatment with 10 µM ABA or 5 µM GA3. Three genes (*ACT11*, *UKN1*and *UKN2*) in combination were used for internal control. A significant difference in comparison with the “Control” was indicated with an asterisk (P<0.05, Statistical significance was determined by one-way analysis of variance (ANOVA) followed by Dunnett's test). Error bars denoted SD.

Since seed germination is governed by two major counteracting phytohormones, ABA and GA, we next investigated whether the expression of *GmMFT* could be affected by ABA or GA. Promoter analysis identified an ABA-responsive element (ABRE) and three GA-responsive elements (GAREs) existed in the putative 3 Kb *GmMFT* promoter (Figure S3). The single ABRE was located on 174–183 bp upstream of the initiation codon. Three separate GAREs were located on 213–219 bp, 2449–2455 bp and 2711–2717 bp upstream of the initiation codon, respectively. The presence of these ABRE and GAREs in *GmMFT* promoter implies that ABA and GA may affect the expression of *GmMFT*. To test this possibility, we monitored the expression change of *GmMFT* in seeds after 12 h or 24 h of imbibition with ABA or GA3 treatment by RT-qPCR (Figure 4). As shown in figure 4A, soybean seeds began to germinate after 12 h of imbibition, this process was significantly inhibited by ABA, while slightly promoted by GA_3_. This result demonstrated that the ABA or GA3 treatment was effective. In this process, the expression level of *GmMFT* was slightly increased in the presence of ABA, but obviously decreased in response to exogenous GA_3_ (Figure 4B).

### Constitutive expression of *GmMFT* does not affect the flowering time in *Arabidopsis*

Since soybean is recalcitrant to genetic transformation, we tried to evaluate the possible role of *GmMFT* in *Arabidopsis* by generating *35S::GmMFT* transgenic plants in both Col and *MFT* null mutant (*mft-2*) background. To test whether *GmMFT* is involved in the regulation of flowering time, the rosette leaf number of *35S::GmMFT* transgenic lines, Col and *mft-2* were counted. The result showed that *GmMFT* did not regulate flowering time in *Arabidopsis*, because there was no significant difference in the total number of rosette leaves among all lines under either long days (LD) or short days (SD) (Figure S4).

### Constitutive expression of *GmMFT* in *Arabidopsis* inhibits the seed germination at the early stage

**Figure 5:**
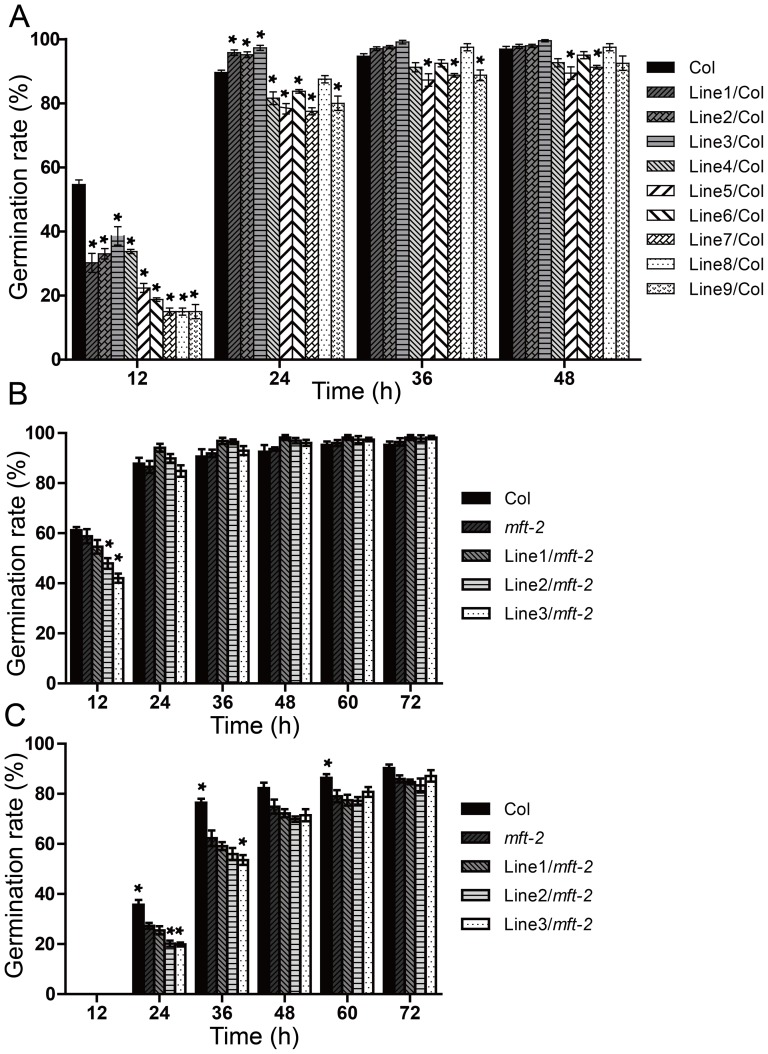
Germination phenotype of *35S::GmMFT* transgenic *Arabidopsis* seeds. A, Germination phenotype of *35S::GmMFT* transgenic lines in Col background on 1/2 MS medium; B, Germination phenotype of *35S::GmMFT* transgenic lines in *mft-2* background on 1/2 MS medium; C, Germination phenotype of *35S::GmMFT* transgenic lines in *mft-2* background on 1/2 MS medium supplemented with 10 µM ABA. A significant difference in comparison with the “Col” for figure 5A or the “*mft-2*” for figure 5B and figure 5C was indicated with an asterisk (P<0.05, Statistical significance was determined by one-way analysis of variance (ANOVA) followed by Dunnett's test). Error bars denoted SEM.

To confirm whether *GmMFT* is involved in the regulation of seed germination, the seed germination rates of different *35S::GmMFT* transgenic lines were compared with that of Col and *mft-2*. The result showed that all nine *GmMFT* overexpressing lines in Col background displayed much lower germination rate than Col at the early stage of seed germination (12 h after transferred to a growth chamber) (Figure 5A). Similarly, two of the three *35S::GmMFT* transgenic lines in *mft-2* background also had lower germination rate than *mft-2* at 12 h after transferred to a growth chamber (Figure 5B). These results suggest that *GmMFT* inhibits the early stage of *Arabidopsis* seed germination. We also analyzed the expression levels of *GmMFT* in the dry seeds of these transgenic lines (Figure S5). It seemed like a correlation between the expression level of *GmMFT* and the germination rate of transgenic lines in *mft-2* background (Figure 5B and Figure S5). However, we did not find a strict correlation between the *GmMFT* expression level and germination rate of transgenic lines in Col background (Figure 5A and Figure S5). Environmental and position effects from transgene insertion may contribute to these fluctuations of *GmMFT* expression levels and to affect the germination rates in transgenic seeds.

Since *MFT* loss-of-function mutant showed hypersensitivity to ABA during seed germination [14], then the ABA effect on germination of *35S::GmMFT* transgenic seeds in *mft-2* background was analyzed (Figure 5C). Consistent with the previous study [14], *mft-2* was more sensitivity to ABA than Col in seed germination. Furthermore, *35S::GmMFT* transgenic line 2 and 3 still had lower germination rate than *mft-2* at the early stage of seed germination, and all three transgenic lines could not rescue the germination phenotype of *mft-2* mutant as *35S::AtMFT* transgenic lines did (Figure 5C) [14].

### *GmMFT* affects the expression of ABA and GA metabolism and signaling genes in *Arabidopsis*

**Figure 6:**
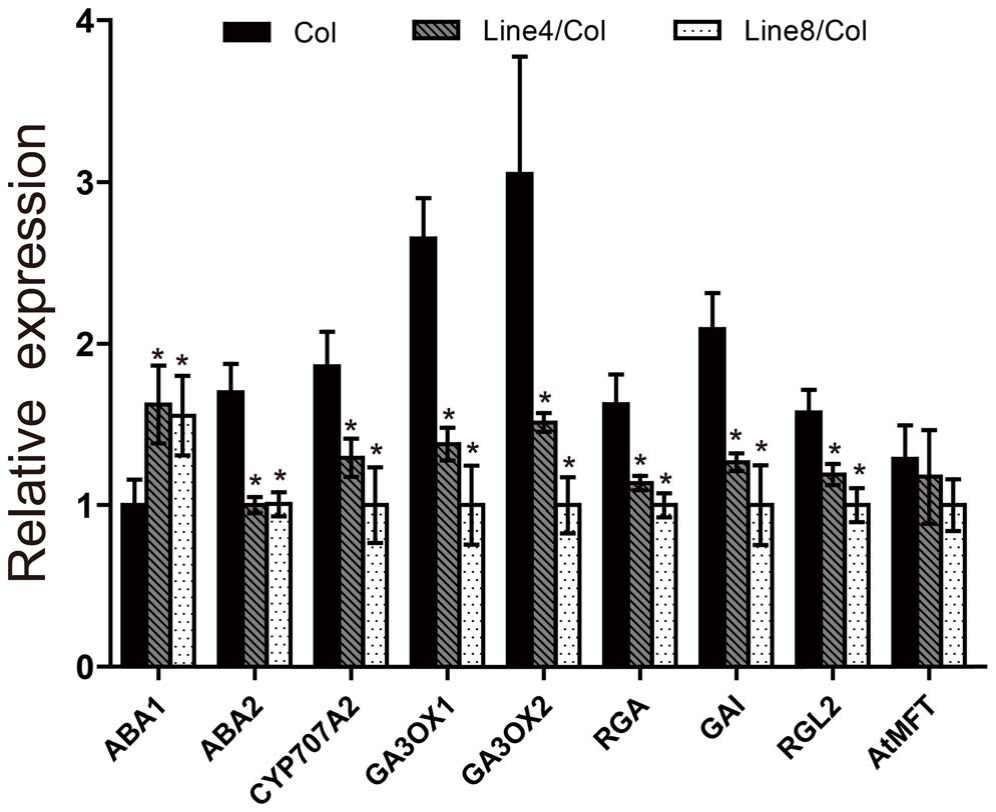
The relative expression of germination-related genes in *35S::GmMFT* transgenic *Arabidopsis* seeds. The seeds at 12-qPCR. Three genes (*CSY3*, At2g20000, At2g04660) in combination were used for internal control. A significant difference in comparison with the “Col” was indicated with an asterisk (P<0.05, Statistical significance was determined by one-way analysis of variance (ANOVA) followed by Dunnett's test). Error bars denoted SD.

To elucidate the possible mechanism of *GmMFT* in inhibiting seed germination, the expression levels of germination-related genes, mainly ABA and GA metabolic or signaling genes, were measured by RT-qPCR in both wild type and *35S::GmMFT* transgenic lines (Figure 6). Among these genes, *ABA1* (an ABA anabolic gene) showed a higher expression level in both two transgenic lines than in Col plants, whereas the transcripts of *ABA2* (an ABA anabolic gene) and *CYP707A2* (a key ABA catabolic gene) were declined in the two transgenic lines, implying that *GmMFT* may be involved in the ABA accumulation in transgenic *Arabidopsis* seeds via modulating the expression of ABA metabolism genes. Moreover, the expression levels of *GA3OX1* and *GA3OX2* (both are involved in GA biosynthesis) decreased significantly in the two transgenic lines, indicating that *GmMFT* may repress GA biosynthesis in transgenic seeds. Taken together, *GmMFT* may inhibit seed germination through the promotion of higher ABA/GA ratio in transgenic seeds. The result also showed that the expression of GA signaling genes, such as *RGA*, *GAI* and *RGL2*, were hampered by the overexpression of *GmMFT*. In addition, we also detected the expression level of *AtMFT* and no obvious change was found in the two transgenic lines compared with wild type, indicating that the overexpression of *GmMFT* didn't influence the expression of *AtMFT* and the effect of *GmMFT* on *Arabidopsis* seed germination was not due to *AtMFT* suppression.

### The N-terminal extension of GmMFT does not contribute to its protein localization

**Figure 7:**
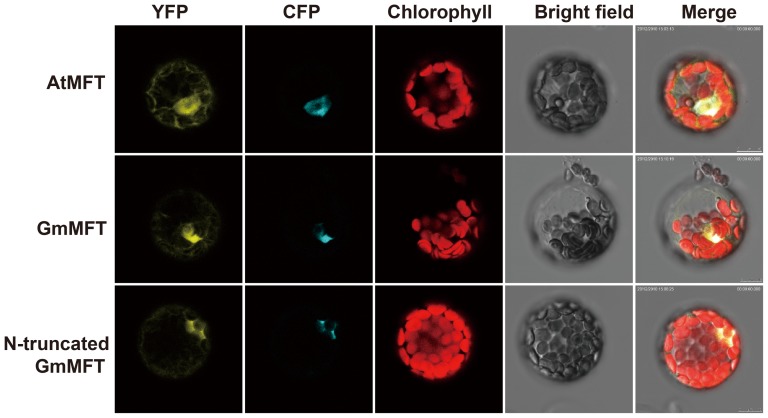
Subcellular localization of GmMFT and N-truncated GmMFT. YFP represents yellow fluorescent signals of GmMFT:YFP, N-truncated GmMFT:YFP or AtMFT:YFP; CFP represents cyan fluorescent signals of the nuclear protein marker AHL22; Chlorophyll represents chloroplast auto-fluorescence; Bright field represents images from white light; Merge represents merge images of the former four images.

The most significant difference between the sequence of GmMFT and AtMFT protein was that GmMFT had an extra 18-amino-acid extension at its N-terminal (Figure 1A), which was predicted as a signal peptide (Figure S1). Therefore, we examined whether this short peptide affects the subcellular localization of GmMFT. A YFP coding sequence was fused to the C-terminal of GmMFT and its truncated form lacking the N-terminal extension, and co-expressed them with a nuclear marker gene *CFP-AHL22* [45] in *Arabidopsis* leaf mesophyll protoplasts. Unexpectedly, this N-extension did not affect the subcellular localization of GmMFT, given by the observation that both full-length and truncated GmMFT distributed in nucleus and cytoplasm as AtMFT did (Figure 7). Even though it, we can't rule out other possibilities that the N-terminal extension confers additional functions on GmMFT.

## Discussion

The PEBP family is evolutionarily conserved across different kingdoms from bacteria to animals and plants [30], [42]. The most thoroughly investigated PEBP members in plants so far are FT-like and TFL1-like proteins [31]–[37]. In *Arabidopsis*, FT and TFL1 act antagonistically to regulate flowering time: FT promotes flowering, while TFL1 represses it. [31], [32], [35]. AtMFT functions as a weak floral inducer, and constitutive expression of it led to slightly early flowering [38]. However, in our present study, the overexpression of *GmMFT* in *Arabidopsis* did not affect the flowering time (Figure S4). Similar results were also observed when *PaMFT1* and *PaMFT2* from *Picea abies* were expressed in *Arabidopsis* [42]. These results imply that GmMFT, as well as PaMFT1 and PaMFT2, may have lost the function on floral induction, or their potentials to promote flowering are much weaker than that of AtMFT. A comparison of their protein sequences has revealed two amino acid residues in AtMFT that differ from those in GmMFT, PaMFT1 and PaMFT2 (Figure S6), suggesting that both residues may be important for distinguishing their functions on flowering control.

Most of the identified *MFT*-like genes in various plant species showed a seed-specific expression pattern, implying a highly conserved function of *MFT*-like genes in seeds [30], [41], [42]. Our expression data of *GmMFT*, which was highly expressed in late stage of developmental seeds (Figure 2, 3A) and declined in early stage of germinating seeds (Figure 3B), also supported part of this hypothesis. The conserved sequences and expression patterns indicated that the *MFT*-like genes may have conserved functions, but they also have undergone functional divergence, especially in terms of regulating seed germination. For example, *AtMFT* has been reported to promote dormancy and germination in freshly matured and after-ripened seeds, respectively [14], [40]. In contrast, *TaMFT* negatively regulated germination not only in immature and freshly matured seeds, but also in after-ripened seeds (1 month after seed maturation) [39]. Based on sequence similarity, GmMFT was more identical to AtMFT than to TaMFT (Figure S2), and was grouped with its orthologous in other dicots, but distant from its orthologous in monocots (Figure 1B). However, the classification of function may be a quite different case, given by the observation that most of the *35S::GmMFT Arabidopsis* transgenic lines showed decreased germination rates in freshly matured (our unpublished data) and after-ripened seeds at the early stage of germination (Figure 5), suggesting that GmMFT is more likely to act as a negative regulator of seed germination as TaMFT did. In *35S::AtMFT* transgenic *Arabidopsis* plants, *AtMFT* promoted germination in after-ripened seeds through a negative feedback loop by modulating ABA signaling [14]. In contrast, *GmMFT* may inhibit germination through the promotion of higher ABA/GA ratio in after-ripened seeds of *35S::GmMFT* transgenic *Arabidopsis* plants, which was supported by the expression changes of genes related to ABA and GA metabolism (Figure 6). But the paradox was the decrease in *RGA*, GAI and *RGL2* expression (Figure 6), given that these genes should be increased when higher ABA/GA ratio in seeds. The result suggests that *GmMFT* can inhibit the expression of *RGA*, GAI and *RGL2* independent of the metabolism of ABA and GA, further hinting that *GmMFT* may also act parallel to the ABA and GA response pathways in regulating seed germination. Interestingly, *AtMFT* is also likely to function parallel to the ABA and GA response pathways in promoting dormancy during seed development [40], implying there is a possibility that *GmMFT* and *AtMFT* share a similar pathway to inhibit germination in after-ripened seeds and to promote dormancy in freshly matured seeds, respectively.

To illuminate the functional variation of *MFT*-like genes in control of seed germination, the protein sequence of GmMFT, TaMFT and AtMFT were analyzed. We found that GmMFT shared some identical amino acid residues with TaMFT, but different from those in AtMFT (Figure 1A), which may be the mutations accumulated evolutionally from the ancestor MFT and beneficial to down-regulate seed germination. In addition, the N-terminal extension of GmMFT may also confer its function opposite to that of AtMFT, even though it did not affect the protein subcellular localization (Figure 7). Detailed sequence shift and residue-substitution experiments among GmMFT, AtMFT, and TaMFT will be helpful to prove these speculations.

Our results showed that GmMFT may be a negative regulator of seed germination. But strictly speaking, we have no direct evidence to demonstrate that *GmMFT* is involved in the regulation of seed germination in soybean due to the limitations of the experimental system. Thus, further research on gain-of-function and loss-of-function of *GmMFT* in soybean should be needed to study the *GmMFT* real function.

## Materials and Methods

### Bioinformatics analysis

Database searches: Protein homolog searches (blastp) using *Arabidopsis* MFT protein was initially conducted against the soybean proteome sequence (phytozome v5.0). Subsequent searches for MFT homologs in phytozome (*Medicago truncatula* and *Oryza sativa*) and GenBank (barley, wheat and maize) were then run.

Proteins alignment and phylogenetic analysis: MFT-like proteins were firstly aligned by clustal W method using MegAlign program, subsequently explored the result as MSF format. The phylogenetic relationships of these proteins were investigated using neighbor-joining (NJ) method in MEGA 4.0 software with 1000 bootstrap replicates.

### Plant materials and growth conditions

The soybean cultivar Kennong 18 (KN18) plants were grown at a temperature 25–28 °C under short day condition (8 h light/16 h dark). The tissues/organs for RT-qPCR and their harvest time were summarized in Table S1. For soybean seed development, the samples were harvested as previously described [44]. The *Arabidopsis* plants were grown at 20–22°C under long day (16 h light/8 h dark) for germination assay and measuring flowering time, or short day (16 h light/8 h dark) for measuring flowering time.

### Seed germination assay

For soybean germination assay: Eighty after-ripened seeds (stored in a dehumidifier cabinet for at least 2 month after harvest) with uniform size and same harvested time were sown on two sheets of filter paper in 12-cm petri dishes, and wetted with 50 ml distilled water. The dishes were incubated in the dark at 25 °C, and then the germinated and ungerminated seeds were counted. The abiotic treatment was carried out at the same condition supplemented with 10 µM ABA (Sigma-Aldrich A1049) or 5 µM GA3 (Sigma-Aldrich G7646). The seeds were scored as germination if the emerging primary root reached 1 mm in length.

For *Arabidopsis* germination assay: Col, *mft-2* and T3 homozygous transgenic seeds were collected at the same time and stored in a dehumidifier cabinet for at least 1 month before the seed germination test was performed. After-ripened seeds were sterilized with 75% (v/v) ethanol for 3 min, and washed with 100% (v/v) ethanol for 2 min. Sterilized seeds were subsequently plated on 1/2 MS medium (Sigma-Aldrich) containing 2% sucrose and 0.8% (w/v) agar. Plates were kept at 4 °C in darkness for 3 days of stratification, and then transferred to a growth chamber set at 22 °C with a 16 h light /8 h dark photoperiod for germination. In the germination assay, at least 50 seeds for each genotype were tested. For ABA treatment, plates contained equal amounts of the corresponding solvents supplemented with 10 µM ABA (Sigma-Aldrich A1049). The seeds were scored as germination if the emergence of the radicle through surrounding structures.

All germination tests were performed on at least three independent replicates, and germination rates were calculated based on the mean ±SD for soybean and mean ±SEM for *Arabidopsis* of three replicates.

### Plasmid construction

To construct *35S::GmMFT* plant expression vector, the full-length coding sequence (CDS) of *GmMFT* was amplified with gene-specific primers (Table S2). The PCR products were subsequently introduced into the entry vector pGWC by TA cloning, and finally transferred to the plant expression vector pLeela via the LR gateway recombination reaction (Invitrogen). To construct the subcellular localization vectors of GmMFT, N-truncated GmMFT and AtMFT, the CDS of them without a terminator codon (TGA) were cloned into the pEXSG–YFP vector using a similar way. The corresponding primers are listed in Table S2.

### RNA extraction and RT-qPCR

Total RNA was extracted using the Trizol Reagent Kit (Invitrogen) according to the manufacturer's instructions. All RNA samples were treated with RNase-free DNase I (Invitrogen) before cDNA synthesis, then reverse transcription reaction was carried out with M-MLV reverse transcriptase (Invitrogen) according to the manufacturer's protocol. The cDNA were diluted and used as the template for RT-qPCR amplification. Two biological replicates of RNA samples were used for downstream applications.

RT-qPCR was performed using an ABI 7300 Real-Time PCR System (Applied Biosystem) and a SYBR Premix Ex Taq™ KIT (Takara) in accordance with the manufacturers' instructions. For the gene expression in soybean, three genes (*ACT11*, *UKN1 *and *UKN2*) in combination were used for internal control [46]. For the gene expression in *Arabidopsis*, *CSY3*, *At2g20000* and *At2g04600* in combination were used for internal control [47]. The method for normalization of RT-qPCR data using three reference genes was performed as previously described [48]. All the primers used for RT-qPCR are listed in Table S2.

### Subcellular localization of GmMFT proteins

The CDS of *GmMFT* without a terminator codon (TGA) was cloned into pEXSG–YFP vector to generate a GmMFT-YFP fusion protein for investigating the subcellular localization of GmMFT in *Arabidopsis* mesophyll protoplasts. pEXSG–GmMFT-YFP vector and pENSG-CFP-AHL22 vector (served as a maker of nuclear localization [45]) were co-transformed into *Arabidopsis* mesophyll protoplasts and the result was recorded by confocal laser scanning microscope (Leica, USA). The same method was used for subcellular localization of AtMFT and N-truncated GmMFT.

## Supporting Information

Figure S1 **Signal peptide prediction of GmMFT.** 1^st^ to 20^th^ amino acid residues consist a signal peptide predicated by an online signal peptide prediction program SignalP (http://www.cbs.dtu.dk/services/SignalP/).
(DOCX)

Figure S2 **Similarity comparison of GmMFT, AtMFT and TaMFT.**
(TIF)

Figure S3 **Promoter analysis of** ***GmMFT*** **.** Putative ABRE and GARE were identified using online software (http://bioinformatics.psb.ugent.be/webtools/plantcare/html/) and marked by green or red inverted triangles respectively. Upstream region, downstream region and introns are represented by white boxes, while exons are indicated by black boxes.
(TIF)

Figure S4 **Flowering phenotypes of** ***35S::GmMFT*** **transgenic lines in the Col or** ***mft-2*** **background**. The total number of rosette leaves at flowering under LD (16 h light/8 h dark) or SD (8 h light/16 h dark) was tested from at least 15 plants for each line.
(TIF)

Figure S5 **The relative expression of** ***GmMFT*** **in different transgenic seeds.** Dry seeds were used for RT-qPCR. At2g20000 was used as internal control.
(TIF)

Figure S6 **Alignment of amino acid sequences of AtMFT, GmMFT, PaMFT1 and PaMFT2.** The red boxes indicate the candidate residues may determine the function of MFT as a floral inducer or not. The sequences are from *Arabidopsis* (AtMFT, At1g18100), *Glycine max* (GmMFT, Glyma05g34030) and *Picea abies* (PaMFT1, AEH59565.1; PaMFT2, AEH59566.1).
(TIF)

Table S1 **Tissues/organs used for RT-qPCR.**
(DOCX)

Table S2 **Primers used in this study.**
(DOCX)
