# Supplementary material for: Identification of a Soybean MOTHER OF FT AND TFL1 Homolog Involved in Regulation of Seed Germination
Source: PLoS One. 2014 Jun 16;9(6):e99642. doi: 10.1371/journal.pone.0099642 (PMC4059689; doi:10.1371/journal.pone.0099642)
Supplement: Figure S1 — Signal peptide prediction of GmMFT. 1st to 20th amino acid residues consist a signal peptide predicated by an online signal peptide prediction program SignalP (http://www.cbs.dtu.dk/services/SignalP/). (DOCX) [file pone.0099642.s001.docx]

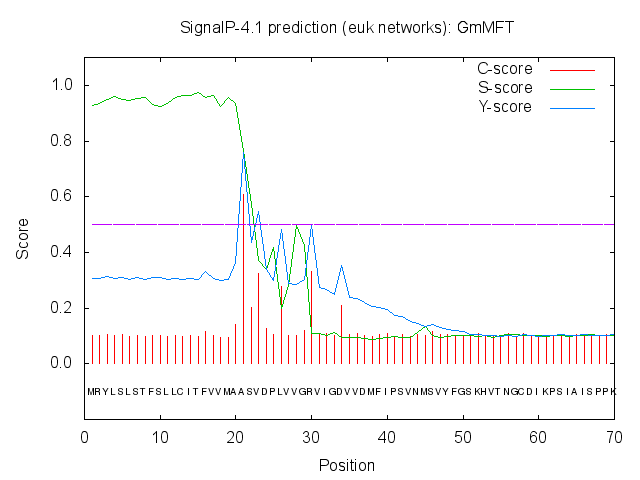


**Note:** *C-score* (raw cleavage site score)

The output from the CS networks, which are trained to distinguish signal peptide cleavage sites from everything else.
Note the position numbering of the cleavage site: the C-score is trained to be high at the position immediately *after* the cleavage site (the first residue in the mature protein).

*S-score* (signal peptide score)

The output from the SP networks, which are trained to distinguish positions within signal peptides from positions in the mature part of the proteins and from proteins without signal peptides.

*Y-score* (combined cleavage site score)

A combination (geometric average) of the C-score and the slope of the S-score, resulting in a better cleavage site prediction than the raw C-score alone. This is due to the fact that multiple high-peaking C-scores can be found in one sequence, where only one is the true cleavage site. The Y-score distinguishes between C-score peaks by choosing the one where the slope of the S-score is steep
