# Supplementary figures and images for: Identification of a Soybean MOTHER OF FT AND TFL1 Homolog Involved in Regulation of Seed Germination
Source: PLoS One. 2014 Jun 16;9(6):e99642. doi: 10.1371/journal.pone.0099642 (PMC4059689; doi:10.1371/journal.pone.0099642)

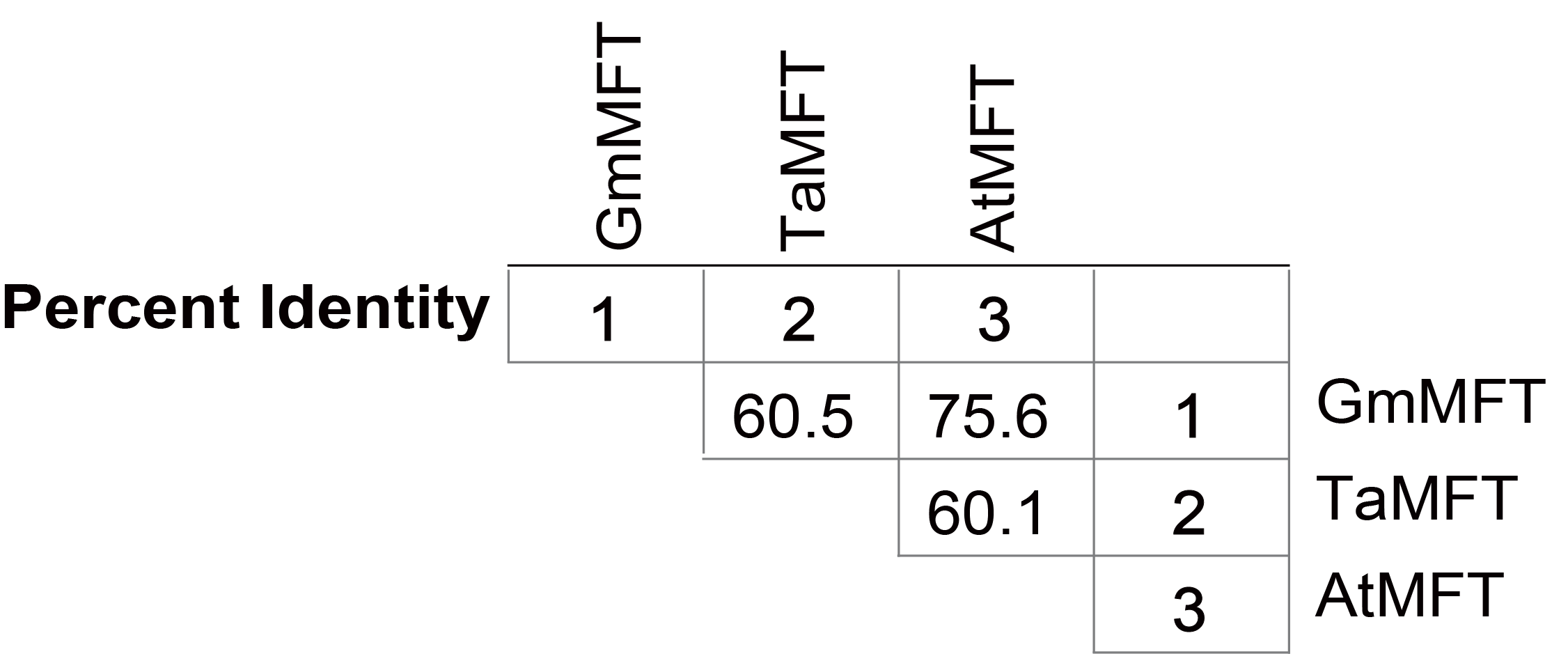

Supplement: Figure S2 — Similarity comparison of GmMFT, AtMFT and TaMFT. (TIF) [file pone.0099642.s002.tif]

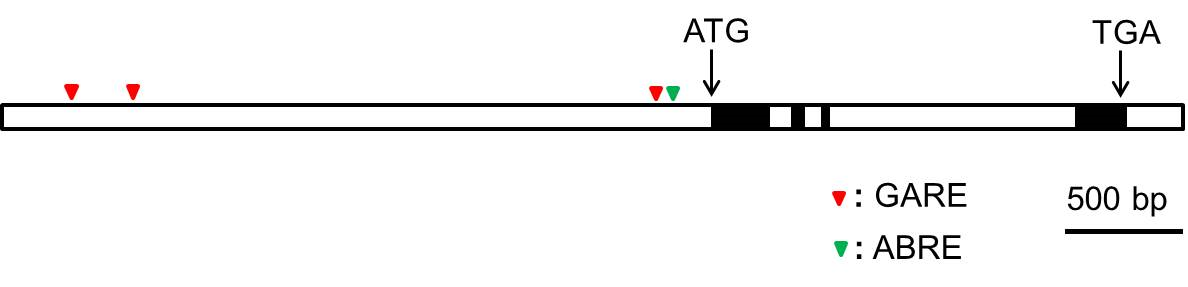

Supplement: Figure S3 — Promoter analysis of GmMFT . Putative ABRE and GARE were identified using online software (http://bioinformatics.psb.ugent.be/webtools/plantcare/html/) and marked by green or red inverted triangles respectively. Upstream region, downstream region and introns are represented by white boxes, while exons are indicated by black boxes. (TIF) [file pone.0099642.s003.tif]

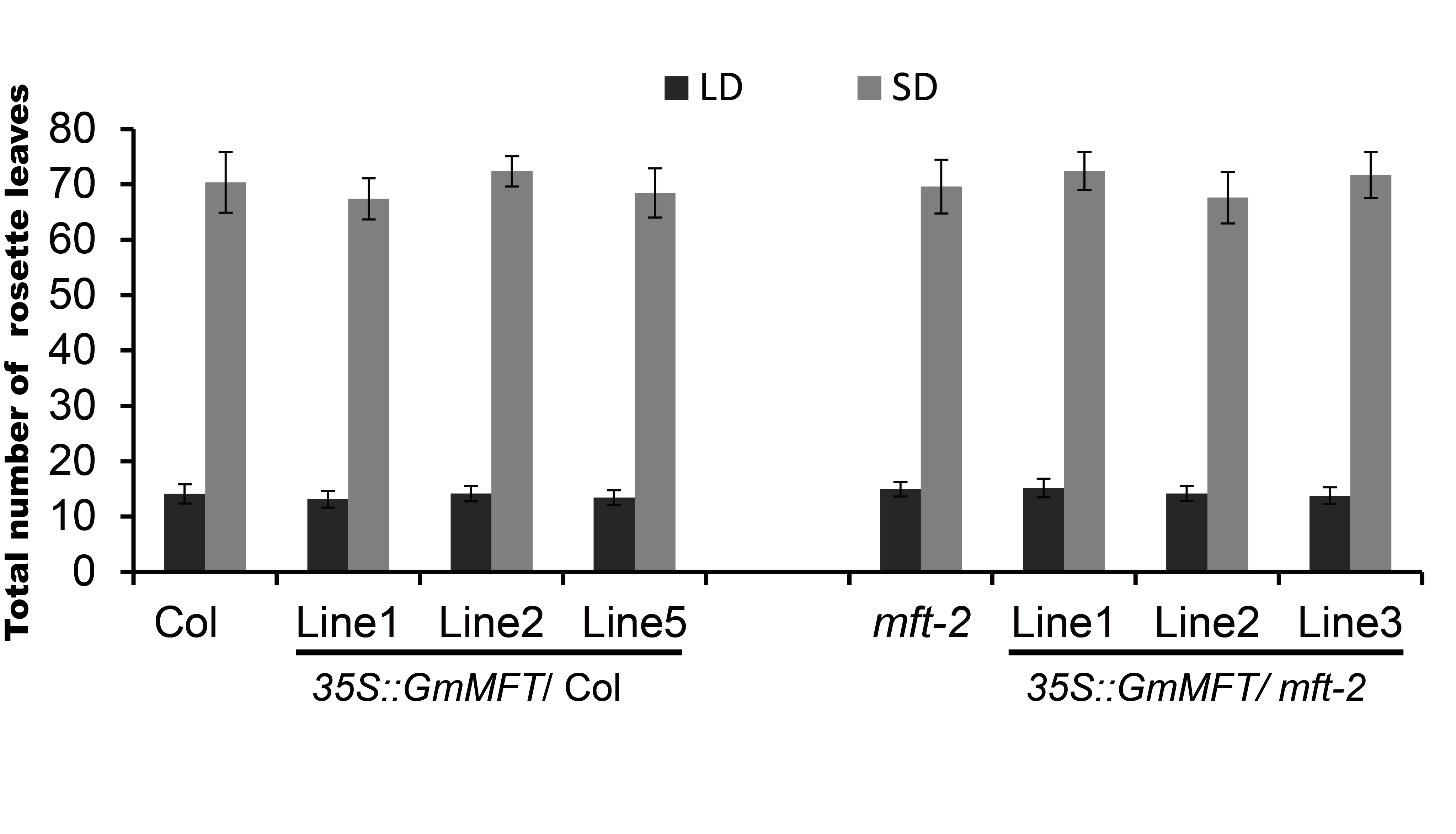

Supplement: Figure S4 — Flowering phenotypes of 35S::GmMFT transgenic lines in the Col or mft-2 background. The total number of rosette leaves at flowering under LD (16 h light/8 h dark) or SD (8 h light/16 h dark) was tested from at least 15 plants for each line. (TIF) [file pone.0099642.s004.tif]

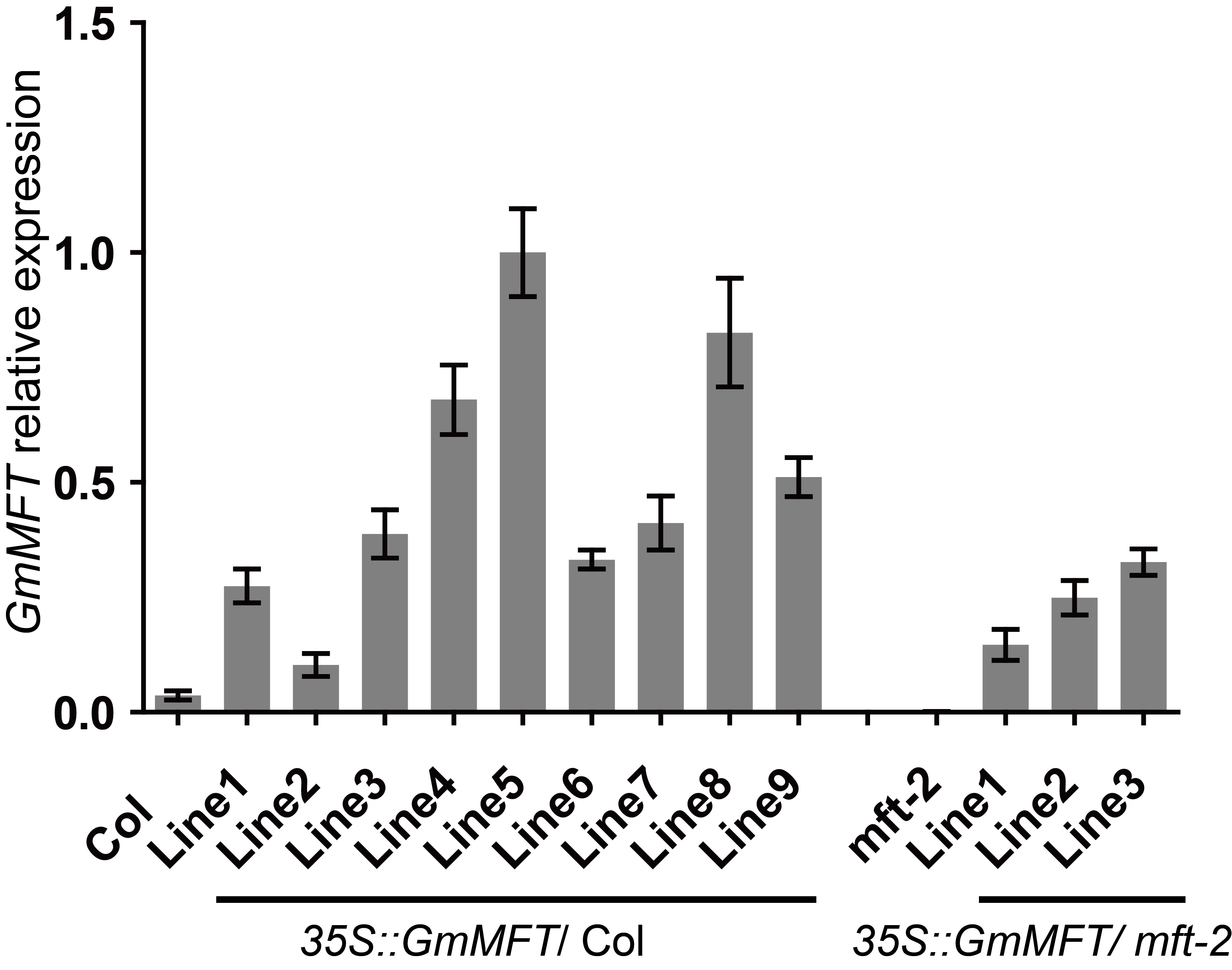

Supplement: Figure S5 — The relative expression of GmMFT in different transgenic seeds. Dry seeds were used for RT-qPCR. At2g20000 was used as internal control. (TIF) [file pone.0099642.s005.tif]

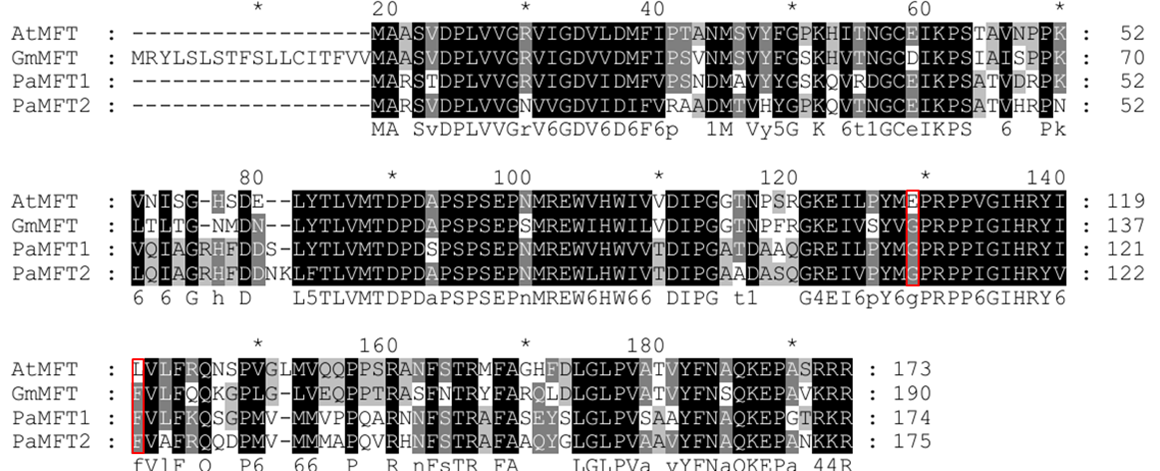

Supplement: Figure S6 — Alignment of amino acid sequences of AtMFT, GmMFT, PaMFT1 and PaMFT2. The red boxes indicate the candidate residues may determine the function of MFT as a floral inducer or not. The sequences are from Arabidopsis (AtMFT, At1g18100), Glycine max (GmMFT, Glyma05g34030) and Picea abies (PaMFT1, AEH59565.1; PaMFT2, AEH59566.1). (TIF) [file pone.0099642.s006.tif]
