# Supplementary material for: Identification of a Soybean MOTHER OF FT AND TFL1 Homolog Involved in Regulation of Seed Germination
Source: PLoS One. 2014 Jun 16;9(6):e99642. doi: 10.1371/journal.pone.0099642 (PMC4059689; doi:10.1371/journal.pone.0099642)
Supplement: Table S1 — Tissues/organs used for RT-qPCR. (DOCX) [file pone.0099642.s007.docx]

| **Stage** | **Tissue/Organ** | **Sample description** |
| --- | --- | --- |
|  | Root | roots when unifoliolates fully opened |
|  | Stem | stems when unifoliolates fully opened |
| Unifoliolate | Cot | cotyledons when unifoliolates fully opened |
|  | U | unifoliolates fully opened |
|  | SA | shoot apex when unifoliolates fully opened |
|  | T1-U | unifoliolates at 1^st^ trifoliolate stage |
|  | T2-U | unifoliolates at 2^nd^ trifoliolate stage |
|  | T3-U | unifoliolates at 3^rd^ trifoliolate stage |
|  | T1-T1 | 1^st^ trifoliolates at 1^st^ trifoliolate stage |
| Trifoliolate | T2-T1 | 1^st^ trifoliolates at 2^nd^ trifoliolate stage |
|  | T3-T1 | 1^st^ trifoliolates at 3^rd^ trifoliolate stage |
|  | T2-T2 | 2^nd^ trifoliolates at 2^nd^ trifoliolate stage |
|  | T3-T2 | 2^nd^ trifoliolates at 3^rd^ trifoliolate stage |
|  | T3-T3 | 3^rd^ trifoliolates at 3^rd^ trifoliolate stage |
|  | Root | roots when onset of flowering |
|  | Stem | stems when onset of flowering |
|  | U | unifoliolates when onset of flowering |
|  | T1 | 1^st^ trifoliolates when onset of flowering |
| Flowering | T2 | 2^nd^ trifoliolates when onset of flowering |
|  | T3 | 3^rd^ trifoliolates when onset of flowering |
|  | T4 | 4^th^ trifoliolates when onset of flowering |
|  | Flower | flowers |
|  | Pod | 40 mm pods in length |
| Podding | Seed | seeds in 40 mm pod in length |
|  | P | 40 mm pod in length without seeds |
