# Supplementary material for: Identification of a Soybean MOTHER OF FT AND TFL1 Homolog Involved in Regulation of Seed Germination
Source: PLoS One. 2014 Jun 16;9(6):e99642. doi: 10.1371/journal.pone.0099642 (PMC4059689; doi:10.1371/journal.pone.0099642)
Supplement: Table S2 — Primers used in this study. (DOCX) [file pone.0099642.s008.docx]

| **Primer** | **Forward sequence** | **Reverse sequence** |
| --- | --- | --- |
| GmMFT | 5' ATGCGTTATTTGTCTCTGAG 3' | 5' TCAGCGCCTCTTAACAGCAG 3' |
| GmMFT (c-fusion) | 5' ATGCGTTATTTGTCTCTGAG 3' | 5' CGGCGCCTCTTAACAGCAG 3' |
| N-truncated GmMFT | 5' ATGGCAGCCTCCGTGGATCCC 3' | 5' CGGCGCCTCTTAACAGCAG 3' |
| qGmMFT | 5' CGTGATCGGCGATGTGGTAG 3' | 5' TTAGGAGGGCTGATGGCAATG 3' |
| qACT11 | 5' ATCTTGACTGAGCGTGGTTATTCC 3' | 5' GCTGGTCCTGGCTGTCTCC 3' |
| qUKN1 | 5' TGGTGCTGCCGCTATTTACTG 3' | 5' GGTGGAAGGAACTGCTAACAATC 3' |
| qUKN2 | 5' GCCTCTGGATACCTGCTCAAG 3' | 5' ACCTCCTCCTCAAACTCCTCTG 3' |
| qABA1 | 5' GGCATTTGGTCTAAGGTGAGAA 3' | 5' CAGACTCGATATCCGCTGGTA 3' |
| qABA2 | 5' TTCTCTTCCTAGTCAAAGGCTTT 3' | 5' GCAGACTTTGGCACCGTGCT 3' |
| qABA3 | 5' CAAAAGGAAGAGTCAAGAGGAAA 3' | 5' TTTCTTTCATCAACTTCACCAGAT 3' |
| qNCED6 | 5' TGAGAGACGAAGAGAAAGAC 3' | 5' GTTCCTTCAACTGATTCTCG 3' |
| qNCED9 | 5' GGAAAACGCCATGATCTCACA 3' | 5' AGGATCCGCCGTTTTAGGAT 3' |
| qAAO3 | 5' GGAGTCAGCGAGGTGGAAGT 3' | 5' TGCTCCTTCGGTCTGTCCTAA 3' |
| qCYP707A1 | 5' TTGGAAAGAGGAGACTAGAG 3' | 5' CACTTGGTGTTTTCTCCTTG 3' |
| qCYP707A2 | 5' AAATGGAGTGCACTCATGTC 3' | 5' CCTTCTTCATCTCCAATCAC 3' |
| qABI3 | 5' ﻿CGGGAGGGACCTGGATGTATT 3' | 5' CCATCACTGGCGGTAATTGAG 3' |
| qABI5 | 5' ﻿CAGCTGCAGGTTCACATTCTG 3' | 5' CACCCTCGCCTCCATTGTTAT 3' |
| qGA20OX1 | 5' GCCTGTAAGAAGCACGGTTTCT 3' | 5' CTCGTGTATTCATGAGCGTCTGA 3' |
| qGA20OX2 | 5' CCCAAGGCTTTCGTTGTCAA 3' | 5' CCGCTCTATGCAAACAGCTCT 3' |
| qGA20OX3 | 5' TCGTGGACAACAAATGGCA 3' | 5' TGAAGGTGTCGCCTATGTTCAC 3' |
| qGA3OX1 | 5' CCCCAACATCACCTCAACTACTGC 3' | 5' CTTCTTCGCTGACCCCAAGTGAAT 3' |
| qGA3OX2 | 5' CCAGCCACCACCTCAAATACTGTG 3' | 5' CTCCCAGTGAACCTAATGCGAACC 3' |
| qGA2OX2 | 5' CCCTCAAATTTTCCGTGAGT 3' | 5' CAGCATTTTACTCAGAGTGTC 3' |
| qRGA | 5' GAATCGAACCATAACGGACC 3' | 5' TTCACAAGCCACCAGATTAC 3' |
| qGAI | 5' GAATCGAACCATAATAGTCCGA 3' | 5' TACCCAAGTAAACCTCCGAC 3' |
| qRGL2 | 5' ﻿GCCCTTACCTGAAGTTCGCTC 3' | 5' TGCATTAAAGCAGGCCATTGC 3' |
| qAtMFT | 5' CGAGCCGAACATGAGAGAAT 3' | 5' AAGTATCTCTTTTCCTCTTGAGGG 3' |
| qAt2g20000 | 5' GTATAGCTCCACCACCACTT 3' | 5' TCTTCTAGGTGCTTGAAGAGT 3' |
| qAt2g04660 | 5' TTCTGGAAGCAGTGGGTGAA 3' | 5' CTCCACTTCCATCTGTAAGC 3' |
| qCSY3 | 5' AGCGCTTTATGGTCCACTTCA 3' | 5' CAACAGTCCCAATCTCTGACAA 3' |
